# Diagnosing a Lie

**Published:** 1917-04

**Authors:** 


**Diagnosing a Lie.** Benuzzi of Graz, by experiments with students, giving them cards marked for truthful and untruthful descriptions, found that the quotient of the inspiratory divided by the expiratory duration was diminished in telling the truth and increased by telling a lie. In other words, it takes more inspiration to tell a lie than the truth.

